# Seroprevalence of *Neospora caninum *in slaughtered native cattle in Kurdistan province, Iran 

**Published:** 2014

**Authors:** Heidar Heidari, Abdolmajid Mohammadzadeh, Jamal Gharekhani

**Affiliations:** 1 *Department of Parasitology, Faculty of Para-Veterinary Sciences, Bu-Ali Sina University, Hamedan, Iran; *; 2 *Department of Microbiology, Faculty of Para-Veterinary Sciences, Bu-Ali Sina University, Hamedan, Iran; *; 3 *Department of Parasitology, Central Veterinary Laboratory, Iranian Veterinary Organization, Hamedan, Iran.*

**Keywords:** Cattle, Iran, Kurdistan, *Neospora caninum*, Seroprevalence

## Abstract

*Neospora caninum* is a worldwide distributed pathogen which causes abortion in cattle leading to economic loss in the cattle industry. The aim of this study was to determine the seroprevalence of *N. caninum *antibodies in the native cattle slaughtered in various areas of Kurdistan province (western Iran) from September 2010 to September 2011. Serum samples from 368 cattle slaughtered in seven slaughterhouses in this region were taken for detection of anti-*N. caninum* antibodies using commercial *N. caninum *ELISA kit. Antibodies to *N. caninum *were found in 29 samples (7.80%). The present study was the first report of *Neospora *infection in this region and indicated that native cattle of Kurdistan province were exposed to this parasite.

## Introduction

*Neospora caninum* is a heteroxenous cyst-forming apicomplexan protozoan which is considered as a major causative of infectious bovine abortion worldwide and has been associated with sporadic, endemic and epidemic abortions.^1^^-^^4^ The infection causes important economic loss to the cattle industry due to reproductive failure associated with abortion and mortality in congenitally infected calves. *Neospora caninum* infection has been reported in dairy cattle herds on all continents.^2^^,^^5^

Dogs and coyotes are the definitive hosts in *N. caninum* life cycle,^6^^-^^8^ whereas cattle and other mammals act as natural intermediate hosts.^9^^,^^10^ In cattle, *N. caninum* infection may occurs by horizontal transmission due to ingestion of sporulated oocysts shed by the definitive host.^11^^,^^12^ However, vertical transmission is the predominant route of infection.^1^^,^^12^ Vertical transmission occurs when tachyzoites cross the placenta of a persistently infected dam and infect the fetus.^13^ Transplacental transmission can occur in consecutive pregnancies in the same cattle and so the infection can persist in cattle herds through many generations.^14^^,^^15^ The infection usually has a chronic course and persists throughout the life of an infected animal.^16^ *Neospora caninum* DNA has been reported in fresh and frozen semen of naturally infected bulls and the possibility of venereal transmission in bovine neosporosis has been suggested.^17^^-^^19^

Non-pregnant adult cattle infected with *N. caninum* do not show any signs of disease, but infection in a pregnant animal may cause abortion, or in the birth of weak, diseased sub-clinically infected or healthy calves.^2^^,^^20^ Abortion may occur at any stage of pregnancy irrespective of whether the infection in the cow is recent, chronic or congenital.^2^^,^^21^

Since recognition of *N. caninum *in 1980, there are only few studies about the seroprevalence of bovine neosporosis in Iran, which are mostly carried out in Tehran^21^, Mashhad north eastern of Iran,^22^^,^^23^ and Kerman south eastern of Iran.^24^ Although there were some reported about sero-prevalence of *N. caninum *in industrial cattle in Iran,^21^^-^^24^ there was no published studies in native cattle. As there was not any published study about seroprevalence of *N. caninum* in native cattle of west part of Iran, this study was performed to determine the prevalence of antibodies to *N. caninum* in native cattle in Kurdistan province, Iran. 

## Materials and Methods

**Study area.** This study was carried out in seven different slaughterhouses of Kurdistan province, western Iran. Kurdistan has an area of about 28,203 Km^2^. According to Iranian Veterinary Organization in 2009, an average of 321,557 native cattle exist in this area.

**Sampling. **Blood samples, were taken randomly from 368 female native cattle of three main geographical zones including seven slaughterhouses of western, central and eastern regions of this province from September 2010 to September 2011. Age of animals was determined according to dental formula and then was matched with owners’ information. All samples were immediately transferred to laboratory of parasitology (Faculty of Veterinary Medicine, Bu-Ali Sina University, Hamedan, Iran). The cattle were divided into four age groups (< 2 years, 2-6 years, 6-8 years and > 8 years old). Blood samples were centrifuged at 1000 *g* for 10 min and then serum was carefully removed. All sera were stored at –20 ˚C until laboratory testing.

Sera were tested for the presence of anti-*N. caninum *antibodies using ELISA Kit (Herdcheck, Maine, USA). According to the manufacturer's instruction, the presence or absence of antibodies to *N. caninum* was determined by sample to positive (S/P) ratio for each sample. Serum samples with S/P ratios less than 0.50 were classified as negative and greater than or equal to 0.50 were classified as positive. All data were analyzed by Chi-square test using SPSS (Version 9.0; SPSS Inc., Chicago, USA). A *p*-value of less than 0.05 was considered statistically significant.

## Results

Anti-*N. caninum* antibodies were detected in 29 of 368 cattle sera (7.80%). The rate of seropositive slaughtered animals in seven different slaughterhouses are presented in Table 1. The difference between prevalence of anti-*N. caninum* antibodies in slaughtered animals in different slaughterhouses was significant (χ^2 ^= 17.492, df = 6,* p *< 0.05). The seroprevalence of anti-*N. caninum* antibodies in different age group are shown in Table 1. There was not an age dependent response as χ^2^ = 0.620, df = 3, *p *= 0.895.

**Table 1:** Seroprevalence of *N. caninum* in relation to age in cattle slaughtered in seven different slaughterhouses. Data are presented as number of samples (percentage of positive samples).

**Slaughterhouses**	**Age group (year)**				
	**≤ 2**	**2-6**	**6-8**	**> 8**	**Total group**
**Sanandaj**	9(11.10%)	28(17.90%)	27(38.60%)	27(11.10%)	91(15.40)
**Bijar**	9(33.30%)	10(0%)	19(1.50%)	44(6.80%)	82(9.80)
**Divandareh**	5(0%)	8(0%)	22(4.50%)	27(3.70%)	62(3.20)
**Marivan**	6(0%)	5(0%)	16(0%)	17(0%)	44(0)
**Dehgolan**	2(0%)	3(33.30%)	3(33.30%)	5(20%)	13(23.10)
**Ghorveh**	5(0%)	5(0%)	12(0%)	16(0%)	38(0)
**Kamyaran**	5(0%)	5(0%)	13(0%)	15(13.30%)	38(5.20)
**Total**	41(9.80%)	64(9.40%)	112(8.00%)	151(6.60%)	368(7.80)

There was no significant difference in the prevalence of abortion between seropositive and non-seropositive cattle (χ^2^ = 0.329, df = 1, *p *= 0.588) indicating that *N. caninum* was not an important causative agent of abortion in the cattle slaughtered in seven different mentioned slaughterhouses in Kurdistan province (Table 2).

**Table 2:** Seroprevalence of *N. caninum* in related to abortion history (χ^2^ = 0.329, *p* = 0.588).

**Abortion history**	**No. of sample (%)**	**No. of positive (%)**
**Yes**	115(31.25)	11(9.56)
**No**	253(68.75)	18(7.11)
**Total**	368(100)	29(7.80)

## Discussion

*Neospora caninum* is considered to be one of the major causative agent of abortion in cattle worldwide.^25^ Several serologic tests including ELISA, immunofluorescent antibody test (IFAT), and direct agglutination test (DAT) were employed to detect anti-*N. caninum *antibodies*.*^2^^,^^24^ At the present, the most commonly serological tests used for the diagnosis of *Neospora* infection are IFAT and ELISA.^24^

The present study showed that the seroprevalence of *N. caninum *infection in the native cattle of Kurdistan province is 7.80%. Our results were nearly similar with the results of investigation on cattle neosporosis of Kars province in Turkey (8.20%).^26^

Antibodies level would be expected to be at its peak level if *N. caninum* were involved in abortion.^27^ Results of the previous studies were in close agreement with findings of the present study because there was no significant difference in the prevalence rate of abortion between seropositive and non-seropositive cattle (*p *= 0.588).^26^^-^^27^ The present study suggested that *N. caninum *was not an important causative agent of abortion in local cattle of Kurdistan province. This finding maybe due to that native breeds are genetically resistant to neosporosis.^2^ In investigation in Brazil on the infection of *N. caninum* in different cattle breeds (Zebu, Holstein, crossbreed Zebu/ Holstein) there was close association between cattle breeds and the frequency of infection by *N. caninum*. The result of this study indicated that breed, which is inherent factor to host, could be considered of high relevance for the distribution and frequency of infection by *N. caninum. *The authors of this study suggested that genetic distance between Holstein and Zebu breeds possibly leads to the significant difference.^28^ These results were similar to finding of a study performed in Ontario which showed that there was a genetic susceptibility to infection by *N. caninum *in Holstein cattle.^29^ Few studies have been carried out considering breed as an epidemiological parameter, to understand the distribution of the infection by *N. caninum* in cattle, probably due to the fact that most of the surveys are restricted to pure breed cattle or to very similar breeds.^29^

In the present study, comparison of *N. caninum* serological status with age groups showed no significant difference (*p *= 0.895). This finding was in close agreement with the result of other studies.^19^^,^^22^ The authors of these studies reported that for most herds seroprevalence levels were equal across all age groups.

In conclusion, the overall seroprevalence of *N. caninum* in native cattle of Kurdistan province was low, suggesting that *N. caninum *was not an important causative agent of abortion in the animals studied in the region. However, further studies on the epidemiological aspect of *N. caninum* infection in cattle and the relationship between abortion in cattle and infection with *N. caninum *in native cattle of this region are required.
